# Australian Consumer Perceptions of Regionally Grown Fruits and Vegetables: Importance, Enablers, and Barriers

**DOI:** 10.3390/ijerph17010063

**Published:** 2019-12-20

**Authors:** Stephanie Godrich, Katherine Kent, Sandra Murray, Stuart Auckland, Johnny Lo, Lauren Blekkenhorst, Beth Penrose, Amanda Devine

**Affiliations:** 1 School of Medical and Health Sciences, Edith Cowan University South West Campus, 585 Robertson Road, Bunbury, WA 6230, Australia; 2 Centre for Rural Health, School of Health Sciences, College of Health and Medicine, University of Tasmania, Newnham Drive, Newnham, Launceston, TAS 7250, Australia; Katherine.kent@utas.edu.au (K.K.); sandra.murray@utas.edu.au (S.M.); Stuart.Auckland@utas.edu.au (S.A.); 3 School of Science, Edith Cowan University, 270 Joondalup Drive, Joondalup, WA 6027, Australia; j.lo@ecu.edu.au; 4 School of Medical and Health Sciences, Edith Cowan University, 270 Joondalup Drive, Joondalup, WA 6027, Australia; l.blekkenhorst@ecu.edu.au (L.B.); a.devine@ecu.edu.au (A.D.); 5 Tasmanian Institute of Agriculture, University of Tasmania, Churchill Avenue, Sandy Bay, Hobart, TAS 7000, Australia; Beth.Penrose@utas.edu.au

**Keywords:** fruit, vegetables, regional, rural, provenance

## Abstract

Fresh fruits and vegetables are a cornerstone of a balanced diet; their consumption has health, environmental, ethical, and economic implications. This pilot study aimed to: (i) measure fruit and vegetable consumption; (ii) understand consumer perceptions of the perceived importance of regionally grown fresh fruit and vegetables (RGFFV); and (iii) identify the barriers and enablers of access and consumption of RGFFV. The study took place in Tasmania (TAS) and South Western Australia (SWA). A 54-item survey included questions relating to purchasing and consumption patterns; barriers and enablers related to access and consumption of RGFFV; and sociodemographic information. Survey data were analyzed using Chi-square test and binary logistic regression. A total of *n* = 120 TAS and *n* = 123 SWA adult respondents participated. SWA respondents had higher intakes of fruit (*p* < 0.001) and vegetables (*p* < 0.001). Almost all respondents (97%) rated purchasing of RGFFV as important. Top enablers included produce freshness (97%), and to financially support local farmers (94%) and the local community (91%). Barriers included limited seasonal availability of the produce (26%), the belief that RGFFV were expensive (12%) and food budgetary constraints (10%). Recommendations include broader marketing and labelling of seasonal RGFFV; increasing ‘buy local’ campaigns; consumer information about how RGFFV benefits producers and communities; and pricing produce according to quality.

## 1. Introduction

Fresh fruits and vegetables are a cornerstone of a balanced diet, and their consumption not only affects human health, but also has major environmental, ethical, and economic implications [1,2,3]. Despite recent efforts of local and state governments to fund campaigns that promote the consumption of in-season, locally sourced, unprocessed, plant-based foods [4], only 5% of Australian adults reportedly consume the recommended levels of both fruit and vegetables [5]. 

Australia produces a wide variety of fresh fruit and vegetables and there are relatively few imports, with more than 90% of fresh fruit and vegetables in supermarkets sourced domestically [6]. Of the fruit and vegetables grown in Australia, only a small proportion of producers are able to take advantage of nearby markets and the low transport costs associated with growing foods close to major capital cities, defined as those with a population of more than 100,000 residents [7]. However, regions that produce high amounts of fruits and vegetables are more likely to have a higher rate of direct-to-consumer sales [8]. These nearby markets in agriculturally productive regions, known in the literature as alternative food networks (AFNs) or civic food networks (CFN), are increasingly present both in societal debates and in scientific literature [9,10,11,12]. The growth of AFNs has reinforced perceptions that food quality is becoming more competitive in food provisioning [13]. These perceptions are integral to understanding the importance of regionally grown food within local food systems and the resultant reconfiguration of producer–consumer interactions. Regionally grown fruits and vegetables are grown, processed, and sold primarily for local or regional markets and they have been conceptualized in terms of scale, provenance, social and supply chain characteristics [14]. In particular, the food supply chain dimension offers the potential to bring consumers closer to the origins of their food through more direct contact with producers [15]. The consumption of regionally grown produce is amongst the fastest-growing food trends, especially in developed countries [16,17,18]. Central to the this trend is the perception that regional food systems are important in their capacity to stimulate a vibrant local economy, where producers and consumers are not separated through a chain of manufacturers, distributors, and retailers [14]. Further, these more localized food systems can offer greater gender equality and social connectedness in communities [19].

International literature suggests that while consumers reportedly value regionally grown produce, their food choices are inconsistent, such as primarily purchasing local products for a special occasion, or when they are discounted [20]. Consumers have also reported mixed perceptions of the price and affordability of local foods [21], though 85% of respondents in a previous study cited cost as a key barrier to consumption [22]. Further, existing evidence suggests limited availability of regionally grown food is an additional barrier [17,23]. Collectively, this evidence suggests budgetary constraints, and to a lesser extent limited availability, appear to be key barriers to purchasing regionally grown produce. With regards to enablers, attitudes and altruistic motives, such as benefiting the local community [17,23,24,25,26,27] and financially supporting local farmers [23,25] have been key reasons cited in previous literature conducted in Australia, South America, and Europe. Personal factors such as the perceived superior quality of produce, taste, a wider range to choose from, as well as enhanced health benefits from regionally grown food have also been reported internationally [17,23,25,26]. European and USA study respondents reported that locally grown produce was of greater quality [23,24,25], and USA participants reported they were fresher and more flavorsome [25]. However, in Australia, there is limited evidence regarding consumer intentions to purchase and consume regionally grown produce. Much of the existing research has focused on food systems as a part of rural development strategies [28,29,30] and distribution [31], which has been primarily viewed from a producer or third-party perspective [32]. The prevailing perspectives in local food research underemphasize the influence that consumers exert on regional food systems [9,10]. Consumer perspectives are required to advance understanding of local food consumption [33]. As published literature has identified inconsistencies across population groups and geographical boundaries, a focused examination of consumer behaviors in unique regions where AFNs may prosper is necessary. The collection of consumer data in two or more similar regions is likely to provide insight into areas of commonality and difference for key success indicators for supporting AFNs in each respective community. Such knowledge may assist local food producers, policymakers, local food advocates, and other professionals seeking to develop local food systems further [34]. Australian consumer evidence to support AFNs is the additional piece of the puzzle required to generate solutions to support increased purchasing of local food and therefore the economic prosperity of local food producers [14]. Therefore, the current gap in the evidence base is a thorough understanding of Australian consumers’ perspective of the importance of locally grown food, in addition to the factors which impede or facilitate consumption of such food. Through a cross-sectional study in two agriculturally productive regions of Australia, this pilot study will provide novel evidence from a consumer perspective, aiming to: (i) measure fruit and vegetable consumption; (ii) understand consumer perceptions of the perceived importance of regionally grown fresh fruit and vegetables (RGFFV); and (iii) identify the barriers and enablers to access and consumption of RGFFV. Given existing literature suggests affordability issues influence purchasing behaviors, we hypothesized that consumers would report a key barrier to consumption of regionally grown fresh fruit and vegetables would be their food budget. Our second hypothesis, also based on previous literature that outlined community and farmer benefits were key drivers to purchasing of regionally grown food, was that consumer intentions to support their local community would be a key motivating factor or enabler of purchasing regionally grown fresh fruit and vegetables. 

## 2. Materials and Methods 

### 2.1. Study Sites

**Figure 1:**
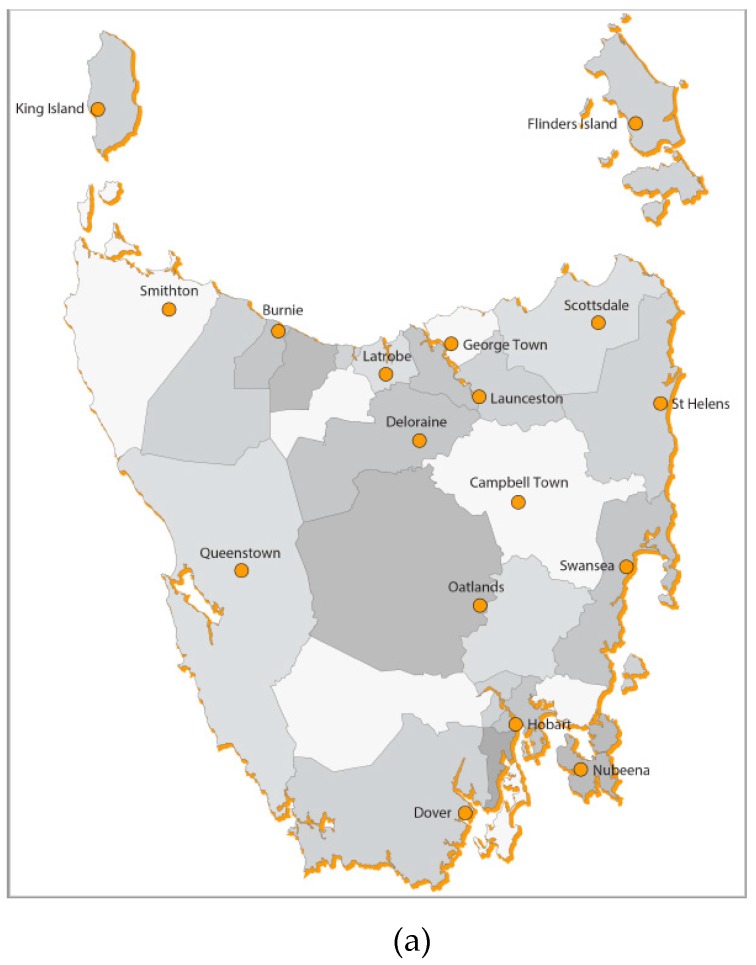

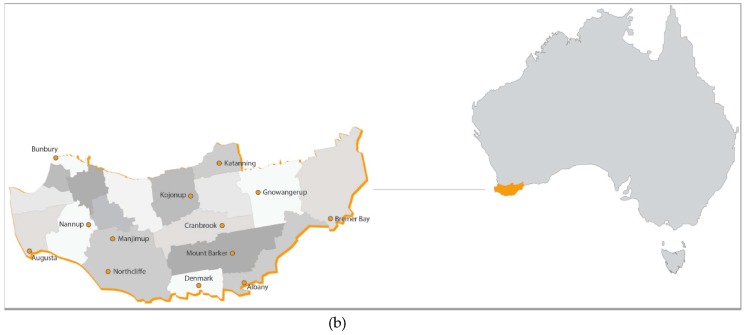
(**a**) Tasmania and (**b**) South Western Australian regions of Australia.

The present cross-sectional study took place in two Australian regions; Tasmania (TAS) and South Western Australia (SWA), which encompassed the South West and Great Southern regions of WA [35] (Figure 1). Whilst recognizing that each region of Australia is unique, TAS and SWA have similar agricultural production in terms of fruit and vegetables when compared to other states and territories. (See Table 1).

### 2.2. Questionnaire Development and Pilot Testing

A 54-item survey was developed by the project team to investigate the importance of RGFFV in SWA and TAS. The survey included specific questions relating to purchasing patterns (*n* = 4); barriers (*n* = 9), and enablers (*n* = 14) relating to access and consumption of RGFFV; and sociodemographic information (*n* = 9) (i.e., age, gender, education). Habitual fruit and vegetable consumption was determined using self-reported daily frequency of fruit and vegetables serves, according to the Australian Guide to Healthy Eating [38]. Participants were asked to rate their perceptions of the importance of purchasing RGFFV with possible responses of ‘very important’, ‘important’, ‘not important’, and ‘not applicable’ [16]. This scale was selected based on a strength-based approach. The primary barrier and enabler questions were based on themes identified in the literature including health, convenience, sensory appeal, quality, price, familiarity, and ethical and social concerns [16,23,39,40,41,42,43,44,45,46,47]. Responses to the level of agreement to each statement were defined along a 5-point Likert scale (‘strongly disagree’, ‘disagree’, ‘neither agree or disagree’, ‘agree’, and ‘strongly agree’).

Lastly, as there are inconsistent definitions of “regionally grown/local food” in the literature [48], participants were asked to select what they felt best described RGFFV from a list of pre-defined options including: fruits and vegetables available at supermarkets, farmers’ markets or roadside stalls, or grown and sold within predefined regions. 

The survey tool was pilot-tested for face and content validity using feedback gathered from various stakeholders, including academics from relevant disciplines (public health nutrition; agriculture) (*n* = 2), a representative from an organization in the food and health sector (*n* = 1), and members of the general community (*n* = 5). The pilot-tested survey tool was amended based on stakeholder feedback.

### 2.3. Data Collection

A cross-sectional study was conducted in TAS and SWA between May and December 2018. Residents of SWA and TAS, aged 18 years and over, were invited to complete either an online or paper-based version of the survey. A-priori sample size calculation was performed at 80% power and 5% level of significance to detect at least a medium effect (Cramer’s V = 0.3) for a Chi-square test between two nominal scales (e.g., importance of RGFFV vs. state). The minimum required sample size was 143 for the two states combined.

Recruitment involved both online and face-to-face strategies through agricultural fairs, markets, small businesses, libraries, and Community Resource Centres. For the paper-based surveys, participants either completed surveys on the spot or returned them using a self-addressed envelope. Participants were provided with a flyer, including a link to an online survey where preferred. Online recruitment strategies included flyer dissemination via local network groups on social media sites; institutional media sites and e-newsletters to university staff and students. Traditional media promotion (i.e., print articles) was also used. 

Online surveys were completed using online survey platforms REDCap electronic data capture tools, hosted at the University of Tasmania (TAS), and Qualtrics software (Version 1019, Copyright 2019, Qualtrics, Provo, UT, USA), used by the Edith Cowan University team (SWA). These were the licensed survey platforms used by the respective universities. Returned hard copy surveys were entered online using REDCap or Qualtrics by two members of the research team (one member for each site). Data were screened by a third member of the team to ensure completeness and minimize outliers. Data were exported from online survey platforms, cleaned, and prepared for statistical analysis. All available survey data was used in the analyses. 

### 2.4. Data Analysis

Data were analyzed using IBM SPSS Statistics for Windows, Version 25.0 (IBM Corp. Armonk, NY). The significance level for all analyses was set at *p* ≤ 0.05. All socio-demographic variables were either categorical or ordinal and were cross-tabulated and summarized with frequencies and proportions. The categories for some of the socio-demographics variables were collapsed due to low counts. Recoded variables included age group, household income bracket, number of adults, and number of children. Age group was reduced to from six categories to five (18–30 years, 31–40 years, 41–50 years, 51–60 years, and 61–75+ years), as the two elderly age groups (61–75 years and 75+ years) were combined. Similarly, household income brackets were reduced from five to four categories (AUD $20,000–40,000, $40,000–60,000, $60,000–80,000, and $80,000–100,000+) with the two top household income brackets combined. Number of adults was reduced from five categories (1, 2, 3, 4, and 5) to three (1, 2, 3, or more). Number of dependents/children was reduced to four categories (0, 1, 2, 3 or more). Chi-square test was used to assess differences in proportions for all socio-demographic variables between the two regions (TAS and SWA).

The fruit and vegetable consumption (serves/day) data were not normally distributed. Mann–Whitney U test was deployed to examine differences in fruit and vegetable consumption between the two regions. The variables of the importance of purchasing RGFFV were re-categorized as ‘important’ (combining ‘very important’ and ‘important’) and ‘not important’. The only ‘not applicable’ response was excluded from the analysis along with other missing responses (*n* = 9). The association between the importance of purchasing RRFFV and socio-demographic variables was assessed using the Chi-square test. A ceiling effect was observed in several of the barrier and enabler questions, and thus response scales were collapsed to allow meaningful analysis. Enabler and barrier questions were each reduced to two categories (‘agree’ or ‘disagree’). Binary logistic regression modelling was used to examine regional differences pertaining to the enabler and barrier questions whilst adjusting for the socio-demographic variables. The final category for each of the socio-demographic variables (in the order specified in Table 2) was set as the reference category. Exploratory factor analysis (EFA), with principal component extraction and equamax rotation, was performed to describe the variability and inter-relationships among the enabler/barrier questions and to identify potential groupings. For each factor, the corresponding variance explained (VE) and factor loadings were described. For ease of interpretation, only loadings >0.5 were noted. 

All subjects gave their informed consent for inclusion before they participated in the study. The study was conducted in accordance with the Declaration of Helsinki, and the protocol was approved by the University of Tasmania’s Social Sciences Human Research Ethics Committee (Project H0017287) with multicenter approval provided by Edith Cowan University’s Human Research Ethics Committee.

## 3. Results

### 3.1. Socio-Demographic Information

Survey data (TAS *n* = 120, SWA *n* = 123) were collated and analyzed. No significant differences were observed between the two regions for age, gender, education, and household income (*p* > 0.05). There were more adults per household (*p* < 0.018) and a greater proportion of main household shoppers in SWA than TAS respondents (95% vs. 75%, *p* < 0.001, see Table 2).

### 3.2. Fruit and Vegetable Consumption

SWA respondents had higher intakes of fruit (*p* < 0.001) and vegetables (*p* < 0.001). Of TAS respondents, 42% self-reported that they consumed one or less servings of fruit per day, whilst 4% of their SWA counterpart reported as such. In terms of vegetable consumption, over 50% of SWA respondents self-reported that they consumed at least the recommended five serves/day, whereas this was only applicable to 31% of TAS respondents.

### 3.3. Importance of Purchasing RGFFV

Almost all respondents (97%) rated purchasing of RGFFV as important. Although a greater proportion of those who were not the main household shoppers (8%) rated it as unimportant compared to the main household shoppers (<2%) (*p* < 0.001), the overwhelming majority believed otherwise. No significant associations were observed between the importance of purchasing RGFFV and other socio-demographic variables (all *p* > 0.05) (Table 3).

### 3.4. Enablers and Barriers for Purchasing RGFFV

**Figure 2:**
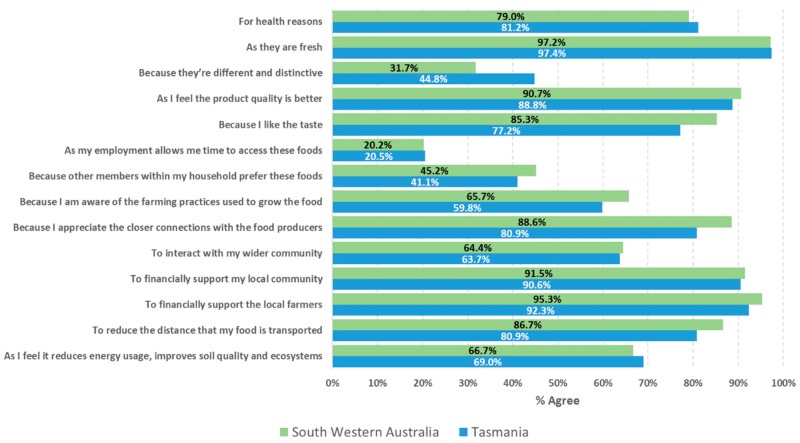
Percentages of agreement from respondents to questions relating to the reasons for choosing to purchase regionally grown fresh fruit and vegetables.

**Figure 3:**
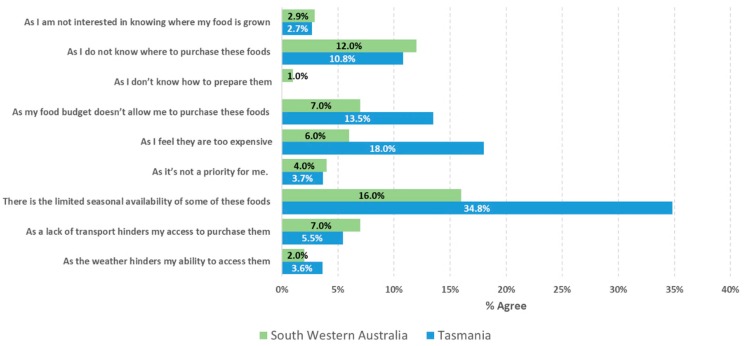
Percentages of agreement from respondents to questions relating to the reasons for NOT choosing to purchase regionally grown fresh fruit and vegetables.

Top enablers of RGFFV cited included freshness of produce (97%), to financially support local farmers (94%) and to support the local community (91%) (Figure 2). The enablers for purchasing RGFFV can be broadly described by 3 factors relating to (1) its impact on the local farmers and community (VE = 20%), (2) personal perception (VE = 18%), and (3) environmental impact (VE = 16%) (Table 4). In contrast, the reasons for ‘not’ choosing to purchase RGFFV were driven by seasonal availability of the produce (26% agreed), the belief that RGFFV were expensive (12% agreed), and food budgetary constraints (10% agreed) (Figure 3). Approximately one in nine respondents (11%) reported that they did not know where to purchase RGFFV. Affordability (VE = 23%), accessibility (VE = 21%), and a lack of interest/knowledge (VE = 20%) were the key factors identified among the barriers (Table 4). There was no difference in opinion between the two regions in relation to these questions (all *p* > 0.05), except for seasonal availability of RGFFV, where 35% of TAS respondents, as compared to 16% of SWA respondents, agreed that this was a barrier to purchase these foods (*p* < 0.007). 

### 3.5. Associations between Enablers/Barriers for Purchasing RGFFV and Respondents’ Socio-Demographic Characteristics

Male respondents were less likely to agree that connections with food producers (OR = 0.3, 95% CI 0.1, 0.8, *p* = 0.017) and the wider community (OR = 0.4, 95% CI 0.2, 0.9, *p* = 0.031) were reasons for purchasing RGFFV than female respondents, and more likely to agree that it is not a priority for them (OR = 15.1, 95% CI 1.4, 163.1, *p* = 0.025). Main household shoppers were more likely to agree that purchasing RGFFV supports the local community (OR = 5.7, 95% CI 1.2, 27.0, *p* = 0.029). The eldest respondents (≥61 years) in the study were more likely to not know where to purchase RGFFV as compared to younger (<40 years) respondents (OR > 6.3, *p* < 0.05). However, the youngest respondents (18–30 years) were more likely to indicate food budget (OR = 8.2, 95% CI 1.2, 55.5, *p* = 0.032) as a reason for not purchasing RGFFV as compared to the eldest group. 

## 4. Discussion

This pilot study is the first of its kind to be conducted in TAS and SWA, and aimed to understand consumption behaviors, consumer perceptions regarding purchasing and consuming RGFFV, and the barriers and enablers of doing so in two agriculturally productive regions of Australia. SWA consumers reported consuming significantly more fruit and vegetables per day, in comparison to TAS consumers. Almost all respondents regarded purchasing RGFFV as important. The primary reasons reported by participants for purchasing and consuming RGFFV included freshness of the produce, to financially support local farmers and the local community. Leading barriers included limited seasonal availability of the produce, the belief that RGFFV were expensive and food budgetary issues. Exploratory factor analysis determined three broad enabler categories: impact on the local farmers and community; personal perception; and environmental impact. The barriers identified related to affordability, accessibility, and a lack of consumer interest/knowledge.

Our findings that TAS respondents consumed less fruit and vegetables than their SWA counterparts is somewhat at odds with previous National Health Survey data: approximately half (52.0%) of TAS adults consumed adequate amounts of fruit, deemed by the Australian Dietary Guidelines as two serves per day [38,49]. This is in contrast to 50.8% of western Australians aged 18 years and above [49] and higher than the Australian average of 51.3% of the adult population consuming two or more serves of fruit per day [50]. According to the Australian Bureau of Statistics, 11.2% of TAS adults aged 18 years and above consumed adequate vegetables [49] in line with the Australian Dietary Guidelines recommendation of five serves per day [50]. However, only 8.9% of WA residents did so [49]. This difference in findings may be explained by different methods of dietary assessment and sample population. This study used a fruit and vegetable specific semi-quantitative food frequency questionnaire and the sample recruited from a range of community events, including agricultural fairs and markets, is less representative then nationally administered surveys [51].

Respondents in our study placed high value on purchasing RGFFV. These findings support previous WA consumer research, where 53% of respondents indicated it was important to them that their store stocked locally grown produce [22]. In addition, 13% of respondents indicated this was the equal most important purchasing factor [22]. 

The enablers of purchasing RGFFV identified by our research supports existing Australian and international evidence, with respect to respondents’ preference for financially supporting local farmers [23]; and supporting the local community [17,23,25,26,27]. For example, participants in Zepeda and Leviten-Reid’s study reported purchasing local food because they were aware of the challenges associated with farming and wanted to support producers [25]. Bianchi et al. (2015) investigated Australian and Chilean respondents’ attitudes to local food and reported a significant positive association between respondents’ attitude towards consuming locally grown food and supporting local food businesses, in both countries’ samples. European research similarly reported increased community benefit as a leading motivator [23]. We also found TAS and SWA consumers were motivated to purchase regionally grown produce for personal reasons, such as perceived increased produce quality, freshness, and taste. This supported existing research [17,25,26,27]. For example, European study respondents perceived greater produce quality was synonymous with locally grown produce [23,24,25], and USA participants reported purchasing locally grown fruit and vegetables due to superior freshness and flavor [25]. However, these findings contrasted other U.S.A research which found almost two-thirds of respondents purchased local produce because of a perceived broader range of products available [26]. Respondents in that study reported that they would purchase their produce elsewhere, should their store experience a shortage of local produce [26]. Interestingly, environmental reasons featured less prominently in our enabler results, particularly with respect to energy use, soil quality, and ecosystems. This was reflected in UK research where less than 4% of consumers reported they chose locally grown produce for environmental reasons, because it was “less harmful for the environment.” [52]. In the USA, more than one quarter (27%) of respondents of a national survey reported environmental concerns, though this was not a key motivator for purchasing local food [53]. These findings collectively suggest that while some consumers are concerned about the environmental impact of their food choices, there is an attitude–behavior gap. 

The limited seasonal availability of local produce was a leading factor in both our research and in existing literature [17,23]. However, this was more prominent for our TAS respondents than SWA respondents. An explanation could be a more limited range of produce in TAS at certain times of the year, as compared to SWA. UK respondents similarly desired out-of-season produce and regarded limited availability as a key barrier to purchasing local produce [23]. Another leading barrier to purchasing and consuming RGFFV reported by our respondents was higher cost, which was likewise reported as a barrier to consumption by 85% of WA respondents in previous research [22] and in a number of international studies [17,23,54]. One study reported cost was cited 103 times (and four times as frequent as other barriers) in their focus groups [54]. Discussion often centered on the key perception that consumers were unable to purchase as many fruit and vegetables as they wanted to, due to the prohibitive cost [54]. In the UK, higher cost reportedly prevented the purchase of local produce [23]. Overall, our findings are consistent with existing literature. While personal factors such as health and taste reasons were important to our participants and other studies’ participants, other results demonstrate that a number of the enablers were “altruistic” in nature [23], whereby consumers valued the contribution that locally-produced food made to their community and local farmers. In our research, consumers did not prioritize environmental factors as key motivators for their purchasing behaviors which is often reported in literature focusing on local produce. Instead, our findings add insight into rural consumers’ perceived importance and rationale for purchasing regionally grown produce relating to people- and community-centered motivations. The findings relating to supporting the local community and farmers is particularly important in the rural context, given the increasing financial pressures the farming sector is experiencing [55]. The findings suggest that rural consumers are becoming increasingly aware of such pressures, perhaps through media coverage, and are choosing to contribute to reducing this burden. 

### 4.1. Education, Policy, Practice, and Research Recommendations

Our findings add strength to existing international and national evidence, and have important implications for policy, practice, and research actors. Consumer education through clearer labelling and promotion of RGFFV is required [21]. For example, extension of ‘buy local’ campaigns to enhance consumer awareness of what is grown seasonally, where it can be purchased and emphasizing its freshness may be important. As a component of such buy local campaigns, food producers should work in collaboration with industry, government, and nutritionists to ensure quick, easy, seasonal fruit and vegetable recipes and tips are widely promoted to consumers. This would increase consumer understanding of seasonal produce available within their locale. 

To increase support for seasonal produce and bolster the economy of local communities, state governments could increase promotion of rural food-producing locations for eco-tourism. An example strategy could include the wider-scale development and promotion of ‘food trails’ featuring and showcasing local food producers in rural locations. However, promotional campaigns should focus on farmer or community benefits rather than focusing on the environmental benefits of regionally grown food, given our findings suggested that this was less of a driver for our respondents. In addition, at the local level, local government authorities (LGA) in WA are in the midst of preparing public health plans, which have a focus on equity and preventative health [56]. LGA could support their local community’s health via ensuring healthy food access features in their public health plan, with a priority on affordable, accessible local food close to transport networks. Governments could also support organizations that sell RGFFV, for example, through food procurement legislation of state-owned food outlets to prioritize inclusion of regional produce. This has been trialed in the USA in schools (e.g., Christensen et al. [57]), military bases (e.g., Dunning et al. [58]) and hospitals (e.g., Kendra et al. [59]), although a number of barriers including negotiating with centralized procurement systems, maintaining food safety and seasonality of supply [58,60] have been identified. Implementing integrated ordering systems, sequencing market entry and growth, and facilitating flexible contracts that work for both the institution/s and the producer/s are recommended for increasing the proportion of RGFFV purchased by these institutions [60].

Previous research has argued the value of producers and retailers informing consumers of how purchasing local food can support local communities [17]. In support of thriving AFNs (or CFNs), consumers have been described as significant change agents in relation to food production. Working to increase the uptake of alternative values in ethical practices of food provisioning is fundamental at a community level [61]. Therefore, greater promotion of producer reinvestment of funds into the local community should occur, such as through promotion of local employment opportunities. 

As price of RGFFV was shown to be a major barrier to consumption of RGFFV in our study, second grade, Grade B or “ugly” fruit and vegetables could be marked at a lower price, which may increase accessibility of RGFFV, increase consumption, and provide producers with a profitable avenue for sale of their lower grade produce. Many of the reasons fruits and vegetables are considered to be second grade are cosmetic or affect shelf life. However, the reasons for price difference and reduction in quality or shelf life must be clearly communicated in order for the reputation of RGFFV or the producer to remain undamaged. In addition, increased clarity and relevant pricing of various fresh produce grades by retailers should occur to promote freshness and quality. Clearer labelling and in-store marketing are two suggested strategies to support this recommendation. Shortening the food supply chain through direct marketing, where growers are able to sell directly to the consumer is also another strategy that could be implemented to reduce costs to the consumer, and therefore increase RGFFV consumption [12].

Capitalizing on educational opportunities is paramount to increase understanding and action on local food systems. To ensure future graduates in the public health nutrition and agriculture disciplines are equipped with the knowledge and skills to promote RGFFV and support AFNs in their communities, universities could incorporate regional and local food systems education into tertiary curricula. In addition, this research conducted in two Australian regions could be expanded and scaled up nationally. A recent systematic review regarding the sustainability of AFNs suggests data is limited and monitoring techniques are needed to understand the value of these alternative systems on the three dimensions of sustainability: social, economic, and environmental [62]. Therefore, further academic contribution could include rigorous measurement of the association between purchasing RGFFV and economic benefits to the local community, in addition to social and environmental dimensions. 

### 4.2. Strengths and Limitations

This study’s strengths included the investigation of consumer perceptions in demographically-similar regions (TAS and SWA) in rural Australia. This facilitated the comparison of research findings between locations, which, to the authors’ knowledge, has not been conducted previously in Australia. The focus on consumer perspectives was also a strength and would inform promotional campaigns to increase purchasing and consumption of regionally grown produce, going beyond previous studies’ focus on producer or third party perspectives. We also based survey questions on existing literature, which increased the study’s quality. Limitations included the use of non-random sampling and a non-validated survey tool in the study settings, although it was assessed for face and content validity. This has implications for the study’s generalizability to other locations, which was compounded by the sample size. This study is also less representative of nationally-administered surveys. Further, the length and complexity of the survey tool, which used a number of matrix questions, may have precluded participation by respondents with lower literacy levels. Another factor potentially skewing participation was the collection of data using both face-to-face and online methods. This approach could have influenced the type of participants who consented to participate and potentially, the results. For example, participants recruited at agricultural fairs may have placed greater importance on regionally grown produce or perceived different barriers and enablers to consumption of such produce. The project was also unfunded, and therefore efforts to maximize participant recruitment were limited by the team members’ time constraints. This study’s novelty would have been enhanced through additional insights into motivators of consumer purchasing behavior. For example, consumer-driven recommendations for specific messaging relating to the altruistic enabler reasons, seasonality or cost-related barriers that this study uncovered.

## 5. Conclusions

Results from our study contributed important Australian findings to the international evidence base regarding consumer perceptions of barriers and enablers to purchasing RGFFV from two agriculturally productive regions of Australia. Findings highlight that opportunities must be harnessed to boost access and consumption of RGFFV, such as increased promotion of seasonal availability in regions through ‘buy local’ campaigns, greater promotion of produce provenance, consumer education through a range of channels to a shift in RGFFV as the preference, and relevant pricing of produce of various quality grades. Collectively, these strategies have the potential to enhance purchasing and consumption of RGFFV in Australia, offering health, social, environmental, and economic benefits for consumers, food system actors, and the wider community. 

## Figures and Tables

**Table 1:** Comparison of agricultural production by region.

Agricultural Production ($m)	WA (Total) [36]	SW WA [37]	TAS [36]
Fruit and nuts (excluding grapes)	217.1	129.4	83.4
Grapes	112.5	41.8	19.0
Vegetables	316.1	101.7	233.1
Total Agriculture	5752.8	-	1078.9

**Table 2:** Characteristics and fruit and vegetable consumption of respondents. Frequencies and proportions, *n* (%), presented.

Variable	Category	TAS	SWA	Overall	*p*-Value
Age in years ^a^	18–30	27 (23.1%)	14 (15.6%)	41 (19.8%)	0.642
	31–40	20 (17.1%)	17 (18.9%)	37 (17.9%)	
	41–50	25 (21.4%)	17 (18.9%)	42 (20.3%)	
	51–60	16 (13.7%)	15 (16.7%)	31 (15.0%)	
	≥61	29 (24.8%)	27 (30%)	56 (27.1%)	
Gender ^a^	Male	36 (30.5%)	20 (22.2%)	56 (26.9%)	0.182
	Female	82 (69.5%)	70 (77.8%)	152 (73.1%)	
Education ^a^	Secondary	21 (17.8%)	24 (26.7%)	45 (21.6%)	0.124
	Tertiary	97 (82.2%)	66 (73.3%)	163 (78.4%)	
Household income ^a^	<20,000–40,000	16 (14.3%)	18 (20.5%)	34 (17.0%)	0.507
	40,000–60,000	16 (14.3%)	16 (18.2%)	32 (16.0%)	
	60,000–80,000	20 (17.9%)	14 (15.9%)	34 (17.0%)	
	80,000–100,000+	60 (53.6%)	40 (45.5%)	100 (50.0%)	
Number of Adults ^a^	1	10 (8.5%)	19 (21.1%)	29 (13.9%)	0.018 *
	2	82 (69.5%)	59 (65.6%)	141 (67.8%)	
	3 or more	26 (22%)	12 (13.3%)	38 (18.3%)	
	mean ± SD	2.2 ± 0.6	2.0 ± 0.7	2.1 ± 0.7	
Number of dependents/children ^a^	0	60 (54.1%)	58 (65.9%)	118 (59.3%)	0.244 *
	1	14 (12.6%)	12 (13.6%)	26 (13.1%)	
	2	26 (23.4%)	13 (14.8%)	39 (19.6%)	
	3 or more	11 (9.9%)	5 (5.7%)	16 (8.0%)	
	mean ± SD	0.9 ± 1.2	0.64 ± 1.1	0.8 ± 1.1	
Main shopper ^a^	Yes	87 (75.0%)	104 (93.7%)	191 (84.1%)	<0.001 **
	No	29 (25.0%)	7 (6.3%)	36 (15.9%)	
Self-reported fruit in serves/day ^b^	1 or less	50 (42.0%)	5 (4.4%)	55 (23.6%)	<0.001 **
	2	42 (35.3%)	34 (29.8%)	76 (32.6%)	
	3	21 (17.6%)	45 (39.5%)	66 (28.3%)	
	4	2 (1.7%)	20 (17.5%)	22 (9.4%)	
	5 or more	4 (3.4%)	10 (8.8%)	14 (6.0%)	
	Median (Q1, Q3)	2 (1, 2)	3 (2, 4)	2 (2, 3)	
Self-reported vegetables in in serves/day ^b^	2	10 (9.1%)	3 (2.6%)	13 (5.8%)	<0.001 **
	3	31 (28.2%)	12 (10.5%)	43 (19.2%)	
	4	32 (29.1%)	37 (32.5%)	69 (30.8%)	
	5	25 (22.7%)	25 (21.9%)	50 (22.3%)	
	6	12 (10.9%)	37 (32.5%)	49 (21.9%)	
	Median (Q1, Q3)	4 (3, 5)	5 (4, 6)	4 (3.25, 5)	

* *p* ≤ 0.05; ** *p* ≤ 0.01; ^a^ Chi-square test; ^b^ Mann–Whitney U-test.

**Table 3:** Importance of purchasing regionally grown fresh fruit and vegetable and demographic variable associations.

Variable	Category	TAS			SWA			Overall		
		*Important*	*Not Important*	*p*-Value	*Important*	*Not Important*	*p*-Value	*Important*	*Not Important*	*p*-Value
Age	18–30	25 (92.6%)	2 (7.4%)	0.533	14 (100%)	0 (0.0%)	0.369	39 (95.1%)	2 (4.9%)	0.692
	31–40	20 (100%)	0 (0.0%)		16 (94.1%)	1 (5.9%)		36 (97.3%)	1 (2.7%)	
	41–50	23 (92.0%)	2 (8.0%)		17 (100%)	0 (0.0%)		40 (95.2%)	2 (4.8%)	
	51–60	16 (100%)	0 (0.0%)		15 (100%)	0 (0.0%)		31 (100%)	0 (0.0%)	
	≥61	28 (96.6%)	1 (3.4%)		26 (100%)	0 (0.0%)		54 (98.2%)	1 (1.8%)	
	Total	112 (95.7%)	5 (4.3%)		88 (98.9%)	1 (1.1%)		200 (97.1%)	6 (2.9%)	
Gender	Male	33 (91.7%)	3 (8.3%)	0.143	19 (100%)	0 (0.0%)	0.600	52 (94.5%)	3 (5.5%)	0.187
	Female	80 (97.6%)	2 (2.4%)		69 (98.6%)	1 (1.4%)		149 (98.0%)	3 (2.0%)	
	Total	113 (95.8%)	5 (4.2%)		88 (98.9%)	1 (1.1%)		201 (97.1%)	6 (2.9%)	
Education	Secondary	21 (100%)	0 (0.0%)	0.288	24 (100%)	0 (0.0%)	0.541	45 (100%)	0 (0%)	0.190
	Tertiary	92 (94.8%)	5 (5.2%)		64 (98.5%)	1 (1.5%)		156 (96.3%)	6 (3.7%)	
	Total	113 (95.8%)	5 (4.2%)		88 (98.9%)	1 (1.1%)		201 (97.1%)	6 (2.9%)	
Household income	<20,000–40,000	16 (100%)	0 (0.0%)	0.266	17 (100%)	0 (0.0%)	0.153	33 (100%)	0 (0.0%)	0.061
	40,000–60,000	15 (93.8%)	1 (6.3%)		16 (100%)	0 (0.0%)		31 (96.9%)	1 (3.1%)	
	60,000–80,000	18 (90.0%)	2 (10.0%)		13 (92.9%)	1 (7.1%)		31 (91.2%)	3 (8.8%)	
	80,000–100,000+	59 (98.3%)	1 (1.7%)		40 (100%)	0 (0.0%)		99 (99.0%)	1 (1.0%)	
	Total	108 (96.4%)	4 (3.6%)		86 (98.9%)	1 (1.1%)		194 (97.5%)	5 (2.5%)	
Main shopper	No	26 (89.7%)	3 (10.3%)	0.065	7 (100%)	0 (0.0%)	0.793	33 (91.7%)	3 (8.3%)	0.021 *
	Yes	85 (97.7%)	2 (2.3%)		102 (99%)	1 (1.0%)		187 (98.4%)	3 (1.6%)	
	Total	111 (95.7%)	5 (4.3%)		109 (99.1%)	1 (0.9%)		220 (97.3%)	6 (2.7%)	

* *p* ≤ 0.05.

**Table 4:** Factor loadings of the enabler and barrier questions. Only loadings >0.5 are displayed. Per cent variance explained by each factor is given in brackets.

**Enabler ^1^**	**Factor 1 (20.1%)**	**Factor 2 (17.7%)**	**Factor 3 (15.6%)**	**Factor 4 (12.9%)**
For health reasons		0.545		
As they are fresh		0.641		
Because they’re different and distinctive				
As I feel the product quality is better		0.831		
Because I like the taste		0.712		
As my employment allows me time to access these foods				0.834
Because other members within my household prefer these foods				0.707
Because I am aware of the farming practices used to grow the food				
Because I appreciate the closer connections with the food producers	0.698			
To interact with my wider community	0.694			
To financially support my local community	0.837			
To financially support the local farmers	0.803			
To reduce the distance that my food is transported			0.843	
As I feel it reduces energy usage, improves soil quality and ecosystems			0.775	
**Barrier ^1^**	**Factor 1 (22.6%)**	**Factor 2 (20.7%)**	**Factor 3 (20.4%)**	**Factor 4 (15.1%)**
As I am not interested in knowing where my food is grown			0.879	
As I do not know where to purchase these foods			0.552	
As I don’t know how to prepare them			0.562	
As my food budget doesn’t allow me to purchase these foods	0.867			
As I feel they are too expensive	0.855			
As it’s not a priority for me.			0.551	
As the limited seasonal availability of some of these foods prevents me from accessing, purchasing and consuming them				0.899
As a lack of transport hinders my access to purchase them		0.851		
As the weather hinders my ability to access them		0.828		

^1^ Kaiser–Meyer–Olkin statistic >0.8, indicating adequate sampling. *p* < 0.001 for Bartlett’s test of sphericity, indicating EFA is suitable for structure detection.
